# Attenuated Humoral Response Against SARS‐CoV‐2 mRNA Vaccination in Allogeneic Stem Cell Transplantation Recipients

**DOI:** 10.1111/cas.15603

**Published:** 2022-09-26

**Authors:** Takashi Toya, Yuya Atsuta, Takahiro Sanada, Tomoko Honda, Daichi Sadato, Noritaka Sekiya, Hiroko Kogure, Sonomi Takakuwa, Daishi Onai, Naoki Shingai, Hiroaki Shimizu, Yuho Najima, Takeshi Kobayashi, Kazuteru Ohashi, Yuka Harada, Michinori Kohara, Noriko Doki

**Affiliations:** ^1^ Hematology Division, Tokyo Metropolitan Cancer and Infectious Diseases Center, Komagome Hospital Tokyo Japan; ^2^ Department of Microbiology and Cell Biology Tokyo Metropolitan Institute of Medical Science Tokyo Japan; ^3^ Clinical Research Support Center Tokyo Metropolitan Cancer and Infectious Diseases Center, Komagome Hospital Tokyo Japan; ^4^ Department of Infection Prevention and Control, Tokyo Metropolitan Cancer and Infectious Diseases Center, Komagome Hospital; ^5^ Department of Clinical Laboratory, Tokyo Metropolitan Cancer and Infectious Diseases Center, Komagome Hospital

**Keywords:** SARS‐CoV‐2, COVID‐19, allogeneic stem cell transplantation, mRNA vaccine, humoral immunity

## Abstract

Antibody persistence several months after SARS‐CoV‐2 mRNA vaccination in allogeneic stem cell transplantation recipients remains largely unknown. We sequentially evaluated the humoral response to two doses of mRNA vaccines in 128 adult recipients and identified the risk factors involved in a poor response. The median interval between stem cell transplantation and vaccination was 2.7 years. The SARS‐CoV‐2 S1 antibody became positive after the second vaccination dose in 87.6% of the recipients, and the median titer was 1,235.4 arbitrary units (AU)/mL. In patients on corticosteroid treatment, the corticosteroid dose inversely correlated with antibody titer. Multivariate analysis identified risk factors for poor peak response such as an interval from stem cell transplantation ≤ 1 year, history of clinically significant cytomegalovirus infection, and use of > 5 mg/day prednisolone at vaccination. Six months after vaccination, the median titer decreased to 185.15 AU/mL, and use of > 5 mg/day prednisolone at vaccination was significantly associated with a poor response. These results indicate that early vaccination after stem cell transplantation (<12 months) and cytomegalovirus infection are risk factors for poor peak response, while steroid use is important for a peak as well as a persistent response. In conclusion, although humoral response is observed in many stem cell transplantation recipients after two doses of vaccination, antibody titers diminish with time, and factors associated with persistence and a peak immunity should be considered separately.

